# Secreted fungal sulfhydryl oxidases: sequence analysis and characterisation of a representative flavin-dependent enzyme from *Aspergillus oryzae*

**DOI:** 10.1186/1471-2091-11-31

**Published:** 2010-08-20

**Authors:** Greta Faccio, Kristiina Kruus, Johanna Buchert, Markku Saloheimo

**Affiliations:** 1 VTT Technical Research Centre of Finland PO Box 1000, 02044 Espoo, Finland

## Abstract

**Background:**

Sulfhydryl oxidases are flavin-dependent enzymes that catalyse the formation of de novo disulfide bonds from free thiol groups, with the reduction of molecular oxygen to hydrogen peroxide. Sulfhydryl oxidases have been investigated in the food industry to remove the burnt flavour of ultraheat-treated milk and are currently studied as potential crosslinking enzymes, aiming at strengthening wheat dough and improving the overall bread quality.

**Results:**

In the present study, potential sulfhydryl oxidases were identified in the publicly available fungal genome sequences and their sequence characteristics were studied. A representative sulfhydryl oxidase from *Aspergillus oryzae*, AoSOX1, was expressed in the fungus *Trichoderma reesei*. AoSOX1 was produced in relatively good yields and was purified and biochemically characterised. The enzyme catalysed the oxidation of thiol-containing compounds like glutathione, D/L-cysteine, beta-mercaptoethanol and DTT. The enzyme had a melting temperature of 57°C, a pH optimum of 7.5 and its enzymatic activity was completely inhibited in the presence of 1 mM ZnSO4.

**Conclusions:**

Eighteen potentially secreted sulfhydryl oxidases were detected in the publicly available fungal genomes analysed and a novel proline-tryptophan dipeptide in the characteristic motif CXXC, where X is any amino acid, was found. A representative protein, AoSOX1 from *A. oryzae*, was produced in *T. reesei *in an active form and had the characteristics of sulfhydryl oxidases. Further testing of the activity on thiol groups within larger peptides and on protein level will be needed to assess the application potential of this enzyme.

## Background

Disulfide bonds are essential for the stability and function of intracellular and secreted proteins. The subject of this work are sulfhydryl oxidases, SOX, and in particular glutathione oxidases (E.C. 1.8.3.3), enzymes catalysing the formation of *de novo *disulfide bonds between thiol groups with the subsequent reduction of oxygen to hydrogen peroxide (equation 1).

(1) $$\text{2R}-\text{SH}+{\text{O}}_{\text{2}}\to \text{R}-\text{S}-\text{S}-\text{R}+{\text{H}}_{\text{2}}{\text{O}}_{\text{2}}$$

The name sulfhydryl oxidase is sometimes also referred to thiol oxidases (EC 1.8.3.2), enzymes that also oxidise thiol groups using oxygen as electron acceptor but reducing it to water. Thiol oxidases have been isolated from fungi, e.g. *Mycothecium *[1], *Piricularia *and *Polyporus *[2].

The first secreted fungal enzyme containing FAD and able to oxidise glutathione and several sulfhydryl compounds was reported in 1982 from *Penicillium sp. *K-6-5. This enzyme had negligible activity on cysteines in proteins. Furthermore, it was not effective on the reactivation of reduced RNase A [3]. In 1987, a secreted sulfhydryl oxidase active on protein associated thiol groups was isolated in *Aspergillus niger *culture filtrates [4]. The enzyme was found to be homodimeric, and each subunit was binding tightly but non-covalently a FAD molecule. *A. niger *sulfhydryl oxidase was active on glutathione and in a lesser extent on homocysteine, DTT, cysteine, a g-glu-cys dipeptide characterised by a carboxylamide bond [5] and its presence increased the rate of reactivation of reduced ribonuclease A [4]. The sulfhydryl oxidase from *A. niger *and *Penicillium *have a different evolutionary origin than the well-characterised intracellular sulfhydryl oxidases, of the Erv family, and are thought to be more related to thioredoxin reductases and pyridine nucleotide flavin disulfide oxidoreductases [3,4,6]. Their physiological role is however still unclear. Metallo-sulfhydryl oxidases containing iron [7] or copper [8] have also been reported.

The action of sulfhydryl oxidases on small thiol-containing compounds and the production of hydrogen peroxide, similarly to the well-known glucose oxidase [9], make sulfhydryl oxidase very attractive for the food industry. Sulfhydryl oxidases can be a valid alternative to the use of chemical additives, such as potassium bromate or ascorbic acid, for the improvement of the strength and handling properties of wheat dough in the baking industry.

The aim of this work was to analyze the putative secreted sulfhydryl oxidases in the publicly available fungal genomes and to produce and biochemically characterise one of the identified enzymes, i.e. AoSOX1 from *Aspergillus oryzae.*

## Results and Discussion

### Analysis of secreted fungal sulfhydryl oxidases

**Figure 1:**
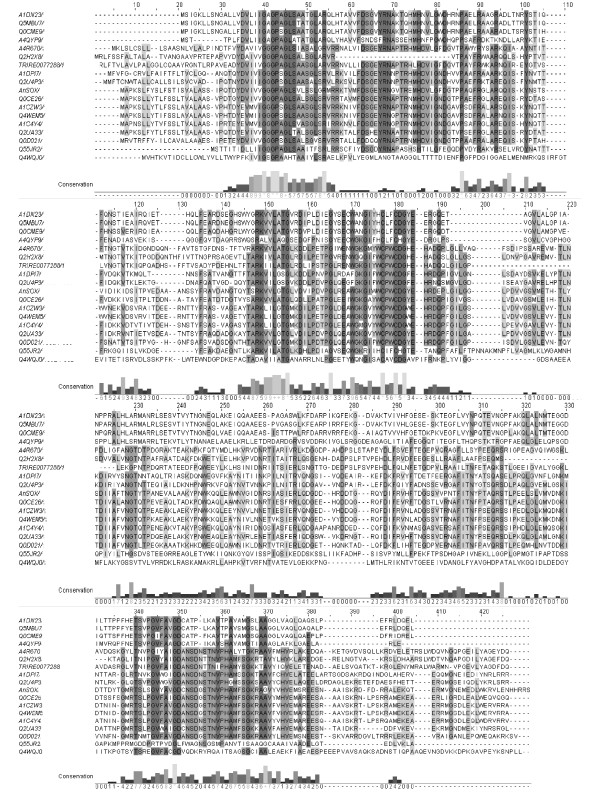
**Sequence alignment**. Sequence alignment of the candidate secreted SOX proteins containing the CXXC motif to the well-known enzyme *A. niger *SOX, [Swiss-Prot:A2QUK3]. Residues background intensity accords to the level of identity and the level of conservation of each residue is indicated by the histogram below and by a 0 to 5 value. The alignment was performed with ClustalW2 and visualised by JalView (http://www.jalview.org). Sequences: [Swiss-Prot:A1DN23] from *N. fischeri*, [Swiss-Prot:Q5MBU7] from *A. fumigatus*, [Swiss-Prot:Q0CME9] from *A. terreus*, [Swiss-Prot:A4QYP9] from *M. grisea*, [Swiss-Prot:A4R670] from *M. grisea*, [Swiss-Prot:Q2H2X8] from *C. globosum*, TRIRE0077288 from *T. reesei*, [Swiss-Prot:A1DPI7] from *N. fischeri*, [Swiss-Prot:Q2U4P3] from *A. oryzae*, AnSOX [Swiss-Prot:A2QUK3] from *A. niger*, [Swiss-Prot:Q0CE26] from *A. terreus*, [Swiss-Prot:A1CZW3] from *N. fischeri*, [Swiss-Prot:Q4WEM5] from *A. fumigatus*, [Swiss-Prot:A1C4Y4] from *A. clavatus*, AoSOX1 [Swiss-Prot:Q2UA33] from *A. oryzae*, [Swiss-Prot:Q0D021] from *A. terreus*, [Swiss-Prot:Q55JR2] from *C. neoformans *and [Swiss-Prot:Q4WQJ0] from *A. fumigatus.*

The search for secreted proteins carrying a predicted disulphide oxidoreductases domain of class II and, in particular, FAD-dependent ones (see Materials and method section) retrieved among 398 proteins 48 with a signal sequence, no ER retention signal and no putative transmembrane segments, and thus are highly likely to be secreted (Table 1). The only characterized protein found among them is the sulfhydryl oxidase from *A. niger *(AnSOX, [NCBI:CAK40401]) [4]. Numerous retrieved proteins have been found in the *Aspergillus spp. *and *Neosartoria fischeri*, a close relative of the *Aspergilli*. Alignment of the sequences identified allowed the selection of 18 proteins possessing the CXXC motif characteristic of thiol:disulfide oxidoreductases like sulfhydryl oxidases (Figure 1). Three main deletions can be identified from the alignment in Figure 1, e.g. protein [Swiss-Prot:Q2H2X8] lacks residues in position 57-83 and 302-318, and protein TRIRE0077288 (http://genome.jgi-psf.org/Trire2/Trire2.home.html) in position 190-211 (residues numbered according to the alignment).

**Table 1:** Secreted fungal sulfhydryl oxidases listed by organism and their key features.

Family	Organism	Name	Status	Length	Signal peptide length	Identity to AnSOX (%)	CXXC motif
*Chaetomiaceae*	*C. globosum*	[Swiss-Prot:Q2H2I0]]	putative	517	26	14.4	--
*Chaetomiaceae*	*C. globosum*	[Swiss-Prot:Q2H2X8]	putative	356	18	37.9	CPWC
*Hypocreaceae*	*T. reesei*	TRIRE0077288^1^	predicted	382	19	37.1	CPWC
*Magnaporthaceae*	*M. grisea*	[Swiss-Prot:A4QYP9]	putative	325	20	24.2	CLFC
*Magnaporthaceae*	*M. grisea*	[Swiss-Prot:A4R670]	predicted	398	19	40.1	CPWC
*Onygenales*	*C. immitis*	[Swiss-Prot:Q1DQ56]	predicted	400	20	10.5	--
*Phaeosphaeriaceae*	*P. nodorum*	[Swiss-Prot:Q0ULD1]	putative	425	18	16.5	--
*Phaeosphaeriaceae*	*P. nodorum*	[Swiss-Prot:Q0V1M0]	predicted	392	22	18.0	--
*Saccharomycetaceae*	*P. stipitis*	[Swiss-Prot:A3LTU5]	predicted	370	20	19.5	--
*Saccharomycetaceae*	*L. elongisporua*	[Swiss-Prot:A5DYY0]	uncharacterised	393	32	14.8	--
*Saccharomycetaceae*	*S. cerevisiae*	[Swiss-Prot:P52923]	evidence at protein level	378	21	17.5	--
*Saccharomycetaceae*	*A. gossypii*	[Swiss-Prot:Q759I7]	predicted	2195	24	3.5	--
*Sordariaceae*	*N. crassa*	[Swiss-Prot:Q7SA02]	predicted	627	22	12.7	--
*Sordariaceae*	*N. crassa*	[Swiss-Prot:Q7SA31]	putative	538	27	12.3	--
*Sordariaceae*	*N. crassa*	[Swiss-Prot:Q7SAD4]	predicted	589	19	12.9	--
*Tremellaceae*	*C. neoformans*	[Swiss-Prot:Q55JR2]	putative	346	21	23.3	CIFC
*Trichocomaceae*	*A. clavatus*	[Swiss-Prot:A1C4Y4]	putative	388	19	61.8	CPWC
*Trichocomaceae*	*A. clavatus*	[Swiss-Prot:A1CE06]	predicted	599	19	9.4	--
*Trichocomaceae*	*N. fischeri*	[Swiss-Prot:A1CZW3]	putative	386	19	62.8	CPWC
*Trichocomaceae*	*N. fischeri*	[Swiss-Prot:A1DD16]	predicted	609	20	10.3	--
*Trichocomaceae*	*N. fischeri*	[Swiss-Prot:A1DJI6]	putative	382	23	14.8	--
*Trichocomaceae*	*N. fischeri*	[Swiss-Prot:A1DKE0]	putative	515	20	11.0	--
*Trichocomaceae*	*N. fischeri*	[Swiss-Prot:A1DN23]	putative	334	28	25.8	CLFC
*Trichocomaceae*	*N. fischeri*	[Swiss-Prot:A1DPI7]	putative	387	23	46.2	CPWC
*Trichocomaceae*	*A. niger*	[Swiss-Prot:A2QUK3]	AnSOX	392	19	100.0	CPWC
*Trichocomaceae*	*A. niger*	[Swiss-Prot:A2R6C0]	predicted	440	23	9.0	--
*Trichocomaceae*	*A. terreus*	[Swiss-Prot:Q0C958]	predicted	360	28	19.4	--
*Trichocomaceae*	*A. terreus*	[Swiss-Prot:Q0CBT8]	predicted	381	21	14.4	--
*Trichocomaceae*	*A. terreus*	[Swiss-Prot:Q0CE26]	predicted	387	19	65.4	CPWC
*Trichocomaceae*	*A. terreus*	[Swiss-Prot:Q0CME9]	putative	335	28	25.8	CLFC
*Trichocomaceae*	*A. terreus*	[Swiss-Prot:Q0CVV0]	predicted	453	21	14.9	--
*Trichocomaceae*	*A. terreus*	[Swiss-Prot:Q0CZE7]	putative	519	48	10.8	--
*Trichocomaceae*	*A. terreus*	[Swiss-Prot:Q0D021]	putative	388	18	55.3	CPWC
*Trichocomaceae*	*A. oryzae*	[Swiss-Prot:Q2TXY9]	predicted	518	41	10.1	--
*Trichocomaceae*	*A. oryzae*	[Swiss-Prot:Q2U4P3]	predicted	389	23	47.3	CPWC
*Trichocomaceae*	*A. oryzae*	[Swiss-Prot:Q2U5L3]	predicted	478	20	14.0	--
*Trichocomaceae*	*A. oryzae*	[Swiss-Prot:Q2UA33]	AoSOX1	384	19	64.7	CPWC
*Trichocomaceae*	*A. oryzae*	[Swiss-Prot:Q2UCA6]	predicted	422	17	9.0	--
*Trichocomaceae*	*A. oryzae*	[Swiss-Prot:Q2UE40]	predicted	625	28	14.7	--
*Trichocomaceae*	*A. oryzae*	[Swiss-Prot:Q2UTD4]	predicted	502	28	12.5	--
*Trichocomaceae*	*A. oryzae*	[Swiss-Prot:Q2UV04]	predicted	513	28	11.8	--
*Trichocomaceae*	*A. fumigatus*	[Swiss-Prot:Q4WEM5]	predicted	386	19	62.3	CPWC
*Trichocomaceae*	*A. fumigatus*	[Swiss-Prot:Q4WF68]	putative	515	20	11.3	--
*Trichocomaceae*	*A. fumigatus*	[Swiss-Prot:Q4WFR5]	putative	382	23	15.8	--
*Trichocomaceae*	*A. fumigatus*	[Swiss-Prot:Q4WLA0]	predicted	508	34	10.1	--
*Trichocomaceae*	*A. fumigatus*	[Swiss-Prot:Q4WQJ0]	inferred from homology	392	29	21.8	CAVC
*Trichocomaceae*	*A. fumigatus*	[Swiss-Prot:Q5MBU7]	predicted	334	27	26.2	CLFC
*Ustilaginaceae*	*U. maydis*	[Swiss-Prot:Q4PCL0]	predicted	583	29	15.0	--

^1 ^*T. reesei *genome, http://genome.jgi-psf.org/Trire2/Trire2.home.html

Sequence features typical of FAD dependent pyridine nucleotide disulphide oxidoreductases (IPR013027) were reflected in three regions. Firstly, a conserved motif characteristic of the Rossmann fold (V/I)(V/I)GXGXXGXXXA/L, where X is any residue, is found in the N-terminal region of the sequences (residues 38-49 in the alignment in Figure 1) suggesting that the proteins bind to a nucleotide cofactor such as FAD or NAD(P), with a βαβ-fold and possibly function as oxidoreductases [10]. The second conserved region is located in the middle of the protein sequence and contains the two conserved cysteine residues of the CXXC motif (residues 177-180 in Figure 1) that is characteristic of the thiol:disulfide oxidoreductases, including sulfhydryl oxidases and, in general, oxidoreductases of the thioredoxin fold family [11]. Many conserved residues are found in this region and the following pattern can be identified RKHHL(A/G)TGXXDX_6_GXX(E/D)XY(G/A)XGXYYCXXC(D/H)GYE (X is any amino acid, H a hydrophobic amino acid and Y an aromatic residue). Here is located the ATG motif common to FAD and NADPH-binding domains and reported to be located at the end of the fourth β-strand interacting with the cofactor [12].

The dipeptide comprised between the cysteine residues has been shown to affect the redox properties of proteins with a CXXC motif [13]. The proline-tryptophan dipeptide found in AoSOX1 and another eleven of the sulfhydryl oxidases studied here has never been characterized, but the N-terminal proline is thought to positively affect the local conformation in DsbA, a protein required for disulfide bond formation in *E. coli *[14]. Quan and co-authors also showed a positive correlation between the presence of an aromatic residue in the C-terminal position and a higher catalytic efficiency. The third conserved region is in the C-terminus and includes firstly, the GD motif (TXHXGHY(A/G)HGD, residues 340-350 in Figure 1) that is involved in the binding of the ribityl moiety of FAD in most flavoproteins with two-dinucleotide binding domains but not common in single FAD-binding domain proteins, e.g. absent in cholesterol oxidase and glucose oxidase, and secondly, the G-helix (AHXXG, residues 362-366 in Figure 1)[12,15]. The final stretch is rich in glutamic acid residues and is predicted to have intrinsic disorder, e.g., after residue E350 in AnSOX and after K349 in AoSOX1 [16].

An overall lower level of conservation is observed in sequence [Swiss-Prot:Q4W4QJ0] from *Aspergillus fumigatus *and [Swiss-Prot:Q55JR2] from *C. neoformans. *Four of the sequences retrieved (at the top of the alignment in Figure 1) clearly group separately and have a different dipeptide (LF) between the cysteine residues of the active site, e.g. [Swiss-Prot:A4QYP9] from *Magnaporthe grisea*, [Swiss-Prot:A1DN23] from *N. fischeri*, [Swiss-Prot:Q0CME9] from *Aspergillus terreus *and [Swiss-Prot:Q5MBU7] from *A. fumigatus*. The three last-named sequences have an unusually long predicted signal peptide, e.g. 28 residues for [Swiss-Prot:Q0CME9] and [Swiss-Prot:A1DN23], 27 residues for [Swiss-Prot:Q5MBU7].

### Production of AoSOX1 in *Trichoderma reesei*

A representative protein, AoSOX1 [NCBI:BAE61582], with 64.7% level of identity to *A. niger *sulfhydryl oxidase was chosen for expression in *T. reesei *and subsequent biochemical characterization. The AoSOX1 protein consists of 384 amino acids including a predicted signal sequence of 19 residues. The closest homologs to AoSOX1 are three putative thioredoxin reductases from *Aspergillus flavus *(99% sequence identity, [NCBI: EED47993]), *Neosartorya fischeri *(70%, [NCBI: XP_001266180]) and *A. fumigatus *(68%, [NCBI: XP_747990]). AoSOX1 gene is predicted to be interrupted by two introns.

The gene coding for AoSOX1 (1304 bp) was amplified by PCR from the genomic DNA of *A. oryzae *and cloned by Gateway recombination into a *T. reesei *expression vector under the control of the strong inducible *cbh1 *promoter. A C-terminal six-histidine tag was added to the expression construct. The expression plasmid was transformed into *T. reesei *and, after screening, one clone, producing the highest activity on glutathione, was selected for enzyme production. The highest production level in shake flask was reached after 5 days of cultivation and was adequate for purification; no fermentor cultures were thus needed. The protein content of the medium at the end of the cultivation was 1.4 g/l and AoSOX1 production level of 70 mg/l accounted for about 5% of the total secreted proteins, as estimated using the specific activity value. The production of AoSOX1 in an active form showed that the two introns were correctly spliced from the transcript. The secretion of AoSOX1 when its gene is expressed in *T. reesei *suggested its extracellular production also in the native strain, *A. oryzae*.

### Enzyme purification

Attempts to purify AoSOX1 with the help of the histidine tag were unsuccessful because of the protein did not bind to copper-chelated Chelating Sepharose Fast Flow resin. Besides, Western blotting analysis of samples containing AoSOX1 with primary anti-Histag antibodies showed no recognition. Furthermore, the C-terminal peptide containing the six-histidine tag was never detected after tryptic digestion and peptide analysis. These findings suggest that possibly the histidine tag was cleaved off the recombinant enzyme by a host protease.

The AoSOX1 enzyme was purified by anion-exchange in a Hitrap DEAE FF column (elution at 166 mM NaCl concentration) and size-exclusion chromatography on a Superdex 75 HR 10/30 column (elution volume 11.3 ml). Results of a typical AoSOX1 purification are summarised in Table 2. AoSOX1 purification by anion exchange and size-exclusion chromatography lead to an activity yield of 68% and a purification factor of 21. The second purification step did not increase the purity but was crucial for the removal of brown-coloured compounds probably derived from the medium used in the cultivations.

**Table 2:** Purification of AoSOX1 from the *T. reesei *culture medium.

	Volume (ml)	**Total activity**^**b **^ **(10**^**6 **^**nkat)**	Total protein (mg)	Specific activity **(10**^**6 **^**nkat mg**^**-1**^**)**	Purification factor	Activity yield (%)
Cell free culture medium^a^	60	311	324	0.96	1	100
Hitrap DEAE FF	108	261	13	20.20	21	84
Superdex 75 HR	8	210	11	19.97	21	68

^a ^The cell-free culture medium (220 ml) was concentrated and buffer exchanged.

^b ^Enzymatic activity was measured with the HVA-peroxidase coupled assay.

### Molecular characterization

**Figure 2:**
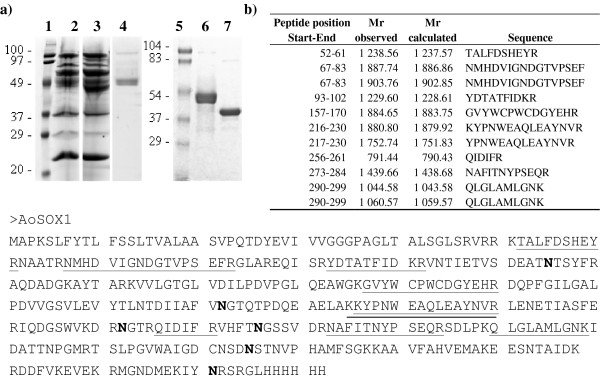
**AoSOX1 molecular characterisation**. (a) SDS PAGE analysis (12%, Tris/HCl) showing the *T. reesei *culture medium of the parental strain (lane 2, 80 μg), of the AoSOX1 expressing strain (lane 3, 80 μg), purified native protein (5 μg and 10 μg,, lane 4 and 6, respectively) and the deglycosylated protein (10 μg, lane 7). Lanes 1 and 5 contain molecular weight markers. (b) Identification by peptide mass fingerprinting of the purified AoSOX1 after in-gel digestion with trypsin. The sequences and masses of the peptides matching with the AoSOX1 sequence are listed and underlined in the protein sequence. Asparagine residues belonging to potential N-glycosylation sites are in bold.

AoSOX1 in the purified form migrates in SDS PAGE in two bands at a molecular weight around 45000 and the MW determined by mass spectrometry was 43959. The difference in molecular mass can be ascribed to N-glycosylation considering that six asparagine residues in the protein are potential N-glycosylation sites and a net mass reduction is observed by SDS PAGE after treatment with PNGase F (Figure 2a, b). Deglycosylation resolved the two bands corresponding to AoSOX1 into a single one of apparently 40000. This suggests that the double band is due to heterogeneity of the N-glycans.

AoSOX1 in the culture medium and in the purified form was identified by peptide mass fingerprinting in the NCBI sequence database (http://www.ncbi.nlm.nih.gov) with a maximum of 24.5% sequence coverage (Figure 2b). Peptides containing putative N-glycosylation sites were not detected suggesting that at least some of the N-glycosylation sites would be occupied by glycans.

### Biochemical characterization

#### Substrate specificity determination and inhibition studies

AoSOX1 showed activity both on small thiol compounds such as cysteine and also on larger molecules such as the tripeptide glutathione (Table 3). The highest activity was registered with glutathione, similarly to the glutathione oxidase from *Penicillium sp*. K-6-5 [3], and the second highest with DTT. A Michaelis-Menten behaviour was observed with all the compounds tested and the highest activity was detected with glutathione as substrate. Glutathione is however an improbable physiological substrate for secreted sulfhydryl oxidases and, even though their role is still unclear, their action on cell-wall or secreted proteins, in the formation of extracellular matrix [17] or for the maturation of peptides produced non-ribosomally [18] cannot be excluded.

**Table 3:** Kinetic constants of AoSOX1 on five different substrates.

Substrate	**K**_**m **_ (mM)	**V**_**max **_ **(nkat ml**^**-1**^**)**	**V**_**max**_**/K**_**m **_ **(s**^**-1**^**)**	**k**_**cat **_ **(s**^**-1**^**)**	Efficacy number **(M**^**-1 **^**s**^**-1**^**)**
Glutathione	2.78 ± 0.56	(15.3 ± 2.89) × 10^7^	55 010	3.60 × 10^6^	1.29 × 10^9^
L-Cys	6.11 ± 0.13	(22.2 ± 0.57) × 10^5^	364	5.23 × 10^4^	8.56 × 10^6^
D-Cys	1.55 ± 0.03	(25.3 ± 0.02) × 10^4^	163	5.95 × 10^3^	3.84 × 10^6^
DTT [thiols]	2.41 ± 0.13	(13.1 ± 0.22) × 10^6^	5 451	3.09 × 10^5^	1.28 × 10^8^
β-mercaptoethanol	9.73 ± 0.08	(21.3 ± 0.05) × 10^5^	219	5.01 × 10^4^	5.15 × 10^6^

AoSOX1 activity was only slightly affected by the presence of the chelating compound EDTA, the salts MgSO_4 _and MnSO_4 _and the denaturing agent urea (Table 4). Similarly to glutathione oxidase from *Penicillium *[3], the activity of AoSOX1 was drastically reduced in presence of ZnSO_4_. This latter result was probably due to the interaction of the zinc ion with cysteine residues necessary for the catalytic activity of AoSOX1. Considering the cysteine content of AoSOX1, e.g. 3 cysteines, there is a high probability that the reactive cysteines are the C177 and C180 forming the CXXC motif.

**Table 4:** Inhibition of AoSOX1 by various compounds.

Inhibitor concentration		**Residual activity**^**a **^**(%)**
EDTA	1 mM	85 ± 5
	10 mM	91 ± 7
KI	1 mM	65 ± 6
	10 mM	48 ± 11
MgSO_4_	1 mM	55 ± 7
	10 mM	80 ± 6
MnSO_4_	1 mM	81 ± 11
	10 mM	94 ± 4
Na_2_SO_4_	1 mM	61 ± 10
	10 mM	70 ± 5
ZnSO_4_	1 mM	1 ± 0
	10 mM	3 ± 1
SDS	1 mM	58 ± 10
	10 mM	64 ± 11
NaCl	1 mM	91 ± 12
	10 mM	74 ± 13
Urea	1 mM	80 ± 6
	10 mM	79 ± 11

^a ^Values are expressed as means ± standard deviations.

#### pH and temperature behaviour

**Figure 3:**
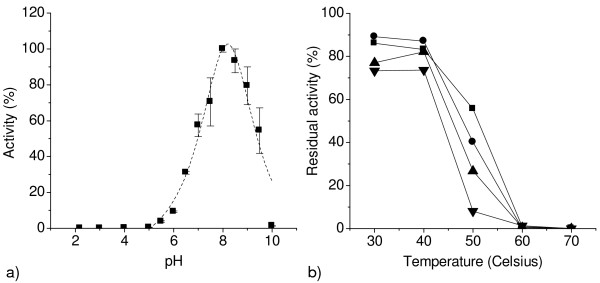
**pH optimum and temperature stability of AoSOX1**. Sulfhydryl oxidase activity was measured with the HVA-peroxidase coupled assay on glutathione 5 mM at room temperature to determine (a) the pH optimum and (b) temperature stability after 30 minutes (filled square), 1 hour (filled circle), 2 hours (filled triangle) and 24 hours (filled inverted triangle) of incubation at different temperatures.

AoSOX1 showed a significant pH stability retaining more than 80% of the initial activity when incubated in a pH range of 4 to 8 after 24 hours (data not shown). AoSOX1 and AnSOX showed a similar pH stability; the pH optimum value of 8, measured for AoSOX1 (Figure 3a) was higher than the value (5.5) reported for AnSOX [4,5] and similar to the ones for glutathione oxidase from *Penicillium *(7-7.8). AoSOX1 showed good temperature stability at 30 and 40°C retaining more than 70% of the initial activity after 24 hours (Figure 3b). The activity of AoSOX1 was reduced to 40% and 8% respectively, after one and 24 hours incubation at 50°C. No activity was detected after 30 minutes of incubation at 60 and 70°C (Figure 3b).

### Molecular properties of AoSOX1

**Figure 4:**
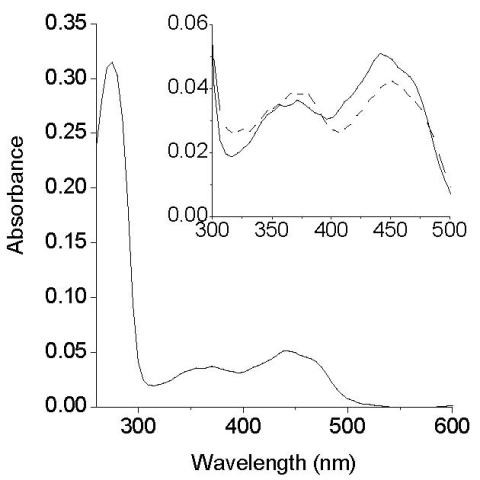
**Absorption spectrum of purified AoSOX1**. Three peaks are visible: at 275 nm, 370 nm and at 440 nm with a shoulder at 465 nm. The inset shows the peaks in visible region before (continuous line) and after denaturation (dotted line); in the latter case the peaks are due to the released FAD cofactor.

The absorption spectrum of AoSOX1 showed three peaks at 275, 370 and 440 nm and a shoulder at 365 nm, like for the glutathione oxidase from *Penicillium *[3], revealing its flavoenzymatic nature (Figure 4). The cofactor was spectrophotometrically identified to be FAD after the cofactor was released from the enzyme by thermal and chemical denaturation (Figure 4, inset).

**Figure 5:**
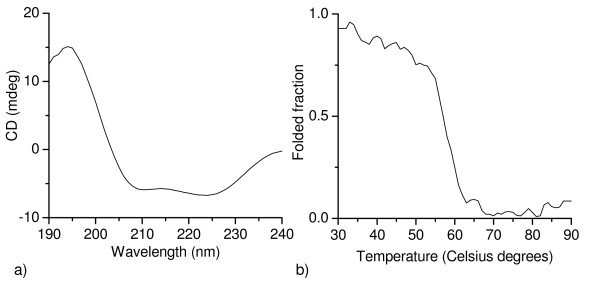
**Spectroscopic analysis of AoSOX1 by circular dichroism**. (a) Spectra in the far-ultraviolet region of AoSOX1 in native form at 25°C and (b) thermal denaturation of AoSOX1 assayed by circular dichroism.

The secondary structure of AoSOX1 presented alpha-helical elements, as evidenced by the two negative peaks at 210 and 225 nm in the CD spectrum (Figure 5a). Thermal denaturation of AoSOX1 revealed a melting temperature of 57°C (Figure 5b). The available three-dimensional structures are of intracellular sulfhydryl oxidases and it would be interesting to see how the structure of a secreted enzyme like AoSOX1 compares with these structures.

**Figure 6:**
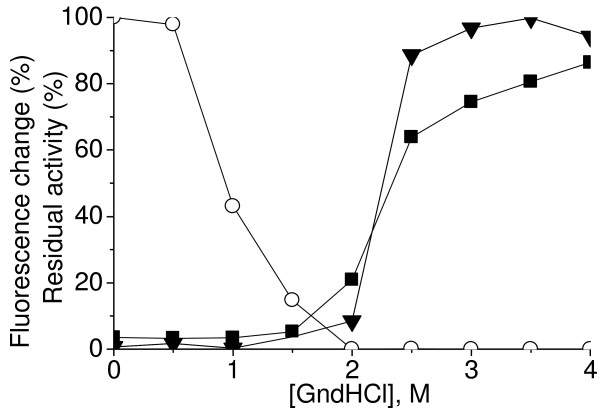
**Equilibrium denaturation curves of AoSOX1**. Intrinsic tryptophan fluorescence (triangle), flavin fluorescence (filled square) and residual activity (empty circle) of AoSOX1 were monitored at increasing guanidinium hydrochloride (GndHCl) concentration.

The stability of AoSOX1 against chaotropic denaturants, e.g. guanidinium hydrochloride, was studied by equilibrium unfolding measurements (Figure 6). The exposure of the tryptophan side chains to a more polar environment, as a result of protein unfolding was reflected in a red shift of the fluorescence peak from 334 to 355 nm and was completed at a 3.5 M denaturant concentration. Tryptophan and FAD fluorescence had both a marked increase between 2 and 2.5 M denaturant concentration that can be due to the presence of three of the six tryptophan residues of AoSOX1 (W154, W160 and W163) in close proximity of the catalytically active di-cysteine pair and thus possibly to the isoalloxazinic ring of the FAD cofactor. A drastic loss of activity was observed at a denaturant concentration above 1 M.

## Conclusions

In the present study the sequences of potentially secreted fungal sulfhydryl oxidases were analyzed and a novel representative enzyme, AoSOX1 from *A. oryzae*, was produced in *T. reesei *in an active form. The characteristics of the enzymes are typical for sulfhydryl oxidases: the enzyme is capable of oxidizing small molecular compounds like glutathione, DTT and cysteine and the enzyme has a non-covalently bound FAD as a cofactor. Further testing of the activity on thiol groups within larger peptides and on protein level will be needed to assess the application potential of this enzyme.

## Methods

All chemicals were purchased from Sigma-Aldrich but D-cysteine hydrochloride monohydrate that was purchased from Fluka. All columns for protein purification were purchased from GE Healthcare and all the purification steps were carried out with an Äkta purifier system (Amersham Biosciences).

### SOX sequence analysis

An in-house database containing 28 public fungal genomes [19] was analysed by InterProScan program [20] for the presence of a pyridine nucleotide disulphide oxidoreductases domain of class II and, in particular, FAD-dependent ones (IPR000103 and IPR013027 respectively). Presence of a target peptide for the secretory pathway was considered and determined with TargetP [21]. Multiple sequence alignments were produced with the software ClustalW [22] and pairwise alignments at EMBL-EBI (http://www.ebi.ac.uk/Tools/emboss/align/index.html).

### Isolation and expression of AoSOX1 gene in *Trichoderma reesei*

The gene coding for AoSOX1 was amplified by PCR from the genomic DNA of *A. oryzae*, strain VTT-D-88348, and with Dynazyme EXT polymerase (Finnzymes, Helsinki, Finland). The PCR program included an initial denaturation step of 3 min at 98°C, followed by 25 cycles of 30 s at 98°C, 30 s at 60°C and 45 s at 72°C. This was followed by a final elongation step of 10 min at 72°C. Cloning was done with the Gateway technology (Invitrogen) and the primers were designed to incorporate *attB *sites in the PCR product to allow the insertion, via BP reaction, into the pDONR221 cloning vector (5'GGGGACAAGT TTGTACAAAA AAGCAGGCTA TCATGGCTCC TAAGTCTCTT TTCTAC3', 3'GGGGACCACT TTGTACAAGA AAGCTGGGTT CAGTGGTGGT GGTGGTGGTG CAGGCCTCTA GACCGATTAT A5'). Primers were also designed to introduce a C-terminal tag of six histidines to the gene product. The subsequent LR recombination reaction transferred AoSOX1 gene into the pMS186 expression vector producing the plasmids pGF008. The pMS186 contained the Gateway reading frame cassette C between the *cbh1 *(cellobiohydrolase 1) promoter and terminator, and a hygromycin resistance cassette. The recombinant plasmid was transformed as described [23] into a variant of the *T. reesei *strain VTT-D-00775 [24]. Transformants were streaked twice consecutively on plates containing hygromycin B (125 μg/ml) and then screened with PCR for the presence of the expression construct. Positive transformants were purified to single spore cultures and grown in shake flasks for 9 days at 28°C in 50 ml of *Trichoderma *minimal medium supplemented with 4% lactose, 2% spent grains and 100 mM piperazine-N,N'-Bis (3-propanesulfonic acid) pH 5.5. SOX activity was assayed after 5, 7 and 9 days on reduced L-glutathione. The transformant giving the highest activity was grown in 250 ml culture medium in a 2 L flask at 28°C for 5 days for routine protein production and subsequent characterisation.

### Activity measurements

#### Oxygen consumption assay

The reaction was initiated by the addition of the enzyme to 1.8 ml of buffered substrate solution in a fully filled-in vial and the oxidation rate (nmol l^-1 ^s^-1^) was calculated from the linear part of the oxygen consumption curve. OXY-10 mini-multi-channel oxygen meter (PreSens Precision Sensing Gmbh, Germany) was used in the measurements.

#### HVA-peroxidase coupled assay

SOX activity was generally measured in a 96 well microtiter plate with a coupled assay modified from Raje [25] where 10 μl of enzyme solution was added to 95 μl of a 1:1 mixture of 1.4 μM peroxidase type II and 1 mM homovanillic acid (HVA); the reaction was started adding 55 μl of substrate, e.g. 5 mM reduced L-glutathione. Reagents were dissolved in 50 mM potassium phosphate buffer, 0.3 mM EDTA pH 7.5. The production of the fluorescent HVA dimer was followed at excitation wavelength 320 nm and emission wavelength 420 nm. The activity as variation of fluorescence was calculated in arbitrary units (AU) per minute. Activity measurements were performed in a black 96-well microtiter plate and fluorescence was measured using a Varioskan spectral scanning multimode reader (Thermo Electron Co., Vantaa, Finland).

**Figure 7:**
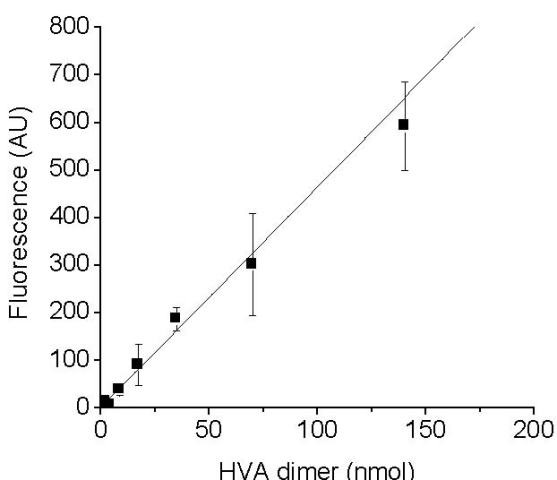
**Conversion of activity values expressed in arbitrary units per minute per ml to katals per ml**. Linear correlation between the moles of HVA-dimer theoretically formed in the reactions at completion and the final fluorescence values measured in arbitrary units (y = 4.63 × +1.40, R = 0.988).

The conversion of activity values in AU to kat ml^-1 ^was possible with a calibration curve (Figure 7). Reaction mixtures containing different amounts of monomeric HVA were monitored to completion and the final fluorescence value was plotted against the number of moles of HVA dimer theoretically produced, and stoichiometrically equivalent to the molecules of oxygen consumed. In example, a standard reaction mixture for activity measurement contained 0.3 mM monomeric HVA that during a standard activity assay could lead to the formation of a maximum of 24 nmoles in dimeric form, in a 160 μl reaction volume. Three identical reaction mixtures were measured, and a conversion factor of 8.6 was obtained for converting the activity values AU ml^-1 ^min^-1 ^to nkat ml^-1^.

The final reaction mixtures were analysed similarly to [26] by HPLC (Hypersil BDS C-18 5 μm, 4.6 × 150 mm, Agilent, operated by a Waters 600 E system controller). The retention time of residual HVA monomer in the reaction mixtures was determined by comparison to solutions of HVA and measuring the absorbance of the eluate at 280 nm (Waters 2996 Photodiode Array detector). The HVA dimer elution was monitored by measuring the fluorescence (λ_ex _= 315 nm, λ_em _= 425 nm, Waters474 Scanning Fluorescence detector). The retention time of HVA in the monomeric form was 2.6 minutes while it was 3.2 minutes for the dimeric form. Dimerization of HVA in the reaction mixtures was confirmed by the appearance of a major fluorescent peak corresponding to dimeric HVA in all reactions.

#### Assay with Ellman's reagent

Ellman's reagent, 5,5'-dithiobis(2-nitrobenzoic acid) (DTNB), was used in a qualitative assay for the detection of SOX activity in the fractions of the purification. The enzyme sample of 10 μl was incubated with 30 μl of 13.3 mM glutathione for 1 hour and the reaction was stopped by adding 300 μl of 0.1 mM DTNB. Reagents were dissolved in PBS buffer (75 mM KH_2_PO_4_, 68 mM NaCl pH 7.5).

### Protein purification

Cell-free medium containing AoSOX1 (220 ml) was concentrated by ultrafiltration (Amicon) using a 10000 cut-off ultrafiltration membrane (Millipore, Espoo, Finland), and was exchanged by dialysis into 20 mM Tris-HCl pH 7 at 4°C. Proteins were separated by anion exchange chromatography (column Hitrap DEAE FF) and eluted in 25 column volumes with a linear 0-300 mM NaCl gradient. Fractions showing a significant activity were then pooled and AoSOX1 was further purified by size-exclusion chromatography (column Superdex 75 HR 10/30, 0.5 ml min^-1 ^flow) in 20 mM Tris-HCl pH 7, 150 mM NaCl.

N-glycans of AoSOX1 were removed from the denatured protein with a PNGase F treatment according to the manufacturer's instructions (Calbiochem, Merck KGaA Darmstadt, Germany) and the result was visualized by SDS PAGE. The protein content was determined with the Bio-Rad DC (Bio-Rad, Richmond, CA, USA) protein assay kit. The purity was estimated to be higher than 95% by electrophoresis in a 12% SDS PAGE [27], using a Pre-stained SDS PAGE Standard (GE Healthcare, Uppsala, Sweden) and Coomassie Brilliant Blue (Pharmacia Biotech, St. Albans, UK). Purified AoSOX1 protein concentration was determined using the extinction coefficient 12160 M^-1 ^cm^-1 ^at 450 nm of the cofactor that was experimentally determined based on the amount of released FAD after SDS (0.2%) and heat treatment (10-30 minutes at 95°C in the dark).

### Spectroscopic methods (circular dichroism, UV/visible and fluorescence spectroscopy)

Absorption spectra were measured in 20 mM Tris-HCl pH 7.0 at 25°C using a Cary Varian 100 Bio UV-Vis spectrophotometer. Circular dichroism (CD) spectra were recorded on a JASCO model J-720 CD spectrometer equipped with a Peltier PTC-38WI thermally controlled cuvette holder. Far-UV CD measurements (190-240 nm) were performed with 2.5 μM purified enzyme in 0.1 M sodium phosphate buffer pH 7 at 25°C, using a 1 mm cell and bandwidth of 1 nm. Spectra were accumulated four times and the values were corrected for buffer contributions. Thermally induced denaturation was followed as the change in a signal at 222 nm in a 30-90°C temperature range with a 2°C min^-1 ^heating rate. Fluorescence spectra were recorded on a Varian Cary Eclipse Fluorescence Spectrophotometer using a 50 μM AoSOX1 solution in 20 mM Tris pH 7 at 20°C. Protein unfolding was done by the addition of amounts of a 6 M guanidinium hydrochloride solution to get a 0 to 5.5 M denaturant concentration in the sample. An incubation time of 15 minutes was allowed before spectrum recording. Excitation wavelength of 290 nm for the tryptophan residues and 450 nm for the flavin cofactor were used, and the fluorescence was recorded in a 300-450 nm and 450-600 nm wavelength intervals, respectively.

### Peptide fingerprinting and molecular weight determination

Bands in the gel (SDS PAGE, 12% acrylamide) were excised and the protein was treated essentially as described by Rosenfeld [28] for peptide mass fingerprinting experiments. The molecular weight of AoSOX1 was determined by MALDI TOF-MS after the enzyme was transferred to distilled water, sinapinic acid was used as matrix in the mass spectrometry. MALDI TOF-MS experiments were carried out in a TOF mass spectrometer Autoflex II (Bruker Daltonik, Bremen, Germany) equipped with laser (160 pulse width, 50 Hz repetition rate) as described [29].

### Determination of pH optimum and stability measurements

The enzymatic activity was measured with the HVA-peroxidase coupled assay; to reduce the influence of pH on the substrate and the reagents of the assay, all solutions and the enzyme were used in a 10-fold higher concentration in assay buffer and diluted to the final concentration in buffer at the proper pH directly in the well of the microtiter plate, e.g. McIlvaine buffer (pH 2.2-8.0), 20 mM Tris/HCl (pH 8.5) and 100 mM CAPSO (pH 8.5-10). AoSOX1 pH stability was determined by incubating the enzyme in McIlvaine buffer (pH 2.2-8.0) and 20 mM Tris/HCl (pH 8.5) and measuring the residual activity after 1 and 24 hours. Temperature stability was assayed incubating aliquots of enzyme solution at 30, 40, 50, 60 and 70°C and measuring the activity in withdrawn samples after 1 and 24 hours with the HVA-peroxidase coupled assay.

### Inhibition studies

Sulfhydryl oxidase activity was measured on glutathione (5 mM) by oxygen consumption assay at room temperature in the presence of inhibitors (EDTA, KI, NaCl, MgSO_4_, MnSO_4_, Na_2_SO_4_, ZnSO_4_, SDS and urea) at 1 and 10 mM concentrations in 50 mM potassium phosphate buffer pH 7.5.

## Authors' contributions

GF carried out the genome mining study, the experimental work and drafted the manuscript. MS and KK participated in the design and conceived of the study and helped to draft the manuscript. JB participated in the design and coordination of the study. All authors read and approved the final manuscript.
